# The impacts of angiotensin converting enzyme inhibitors and angiotensin receptor blockers on rates of acute kidney injury in hospitalized adults receiving multiple nephrotoxins

**DOI:** 10.1186/s12882-026-04884-3

**Published:** 2026-03-26

**Authors:** Michael Tabet, Mary Vaughan-Sarrazin, Brian C. Lund, Spyridon Fortis, Diana Jalal, Jason Misurac, Benjamin R. Griffin

**Affiliations:** 1 University of Iowa Hospitals and Clinics-Internal Medicine, 200 Hawkins Drive, Iowa City, IA 52242 USA; 2 Center for Access & Delivery Research and Evaluation (CADRE), Iowa City Veterans’ Affairs Health Care System, Iowa City, IA USA; 3 Stead Family Department of Pediatrics, University of Iowa Carver College of Medicine, Iowa City, IA USA

**Keywords:** Nephrotoxicity, Angiotensin converting enzyme inhibitors, Angiotensin receptor blockers, Acute kidney injury

## Abstract

**Background:**

Discontinuation of angiotensin converting enzyme inhibitors and angiotensin receptor blockers (ACEI/ARBs) is a common component of many nephrotoxin-associated acute kidney injury (NA-AKI) prevention strategies, though their intrinsic nephrotoxic potential has been increasingly challenged. We hypothesized that ACEI/ARBs would associate with increased overall AKI and stage 2/3 AKI in hospitalized adults receiving multiple nephrotoxins.

**Methods:**

This retrospective cohort study included adult patients admitted to the University of Iowa Hospital for ≥ 3 days from 2014 to 2022. We compared patients receiving 2 nephrotoxins to patients receiving 3, with one being an ACEI/ARB. This allowed us to compare patients not counted as having a high nephrotoxin exposure to a group that would due to an additional ACEI/ARB. The primary outcome was AKI within 7 days of initial exposure. Secondary outcomes included stage 2/3 AKI and AKI occurring within 3 and 14 days of initial exposure. Generalized estimating equations with robust standard errors were used to estimate associations and incorporated inverse probability of treatment weighting (IPTW) based on 30 clinical covariates.

**Results:**

The primary analysis included 15,438 patients exposed to two non-ACEI/ARB nephrotoxins, of which 1,106 (7.2%) also received an ACEI/ARB. Groups were well-matched after IPTW. Adjusted risk of AKI was 25% (95% CI 22–29%) in the ACEI/ARB group, and 20% (19–21%) in the comparator group (adjusted odds ratio 1.35, 95% CI 1.07–1.71). In contrast, ACEI/ARB exposure was not associated with increased risk of stage 2/3 AKI.

**Conclusions:**

In a cohort of hospitalized patients receiving multiple nephrotoxic agents, ACEI/ARBs were associated with higher risks of AKI, but not higher rates of stage 2/3 AKI.

**Supplementary Information:**

The online version contains supplementary material available at 10.1186/s12882-026-04884-3.

## Introduction

Acute kidney injury (AKI) is a major contributor to short- and long-term morbidity and mortality [1, 2], and occurs in 20–25% of hospitalized adults. Many cases of in-hospital AKI feature exposure to at least one medication with nephrotoxic potential [3], and since medication prescribing is a modifiable risk factor, recent studies have focused on prevention of nephrotoxin-associated AKI (NA-AKI) through changes to drug regimens in high-risk patients.

One such prevention program, the Nephrotoxic Injury Negated by Just-in Time Action (NINJA) program, features an intervention for patients receiving ≥ 3 medications with nephrotoxic potential on the same hospital day, and was shown in a large multi-center study to reduce rates of NA-AKI by 24% [4]. It has since spread from the general wards to other pediatric settings including cancer units and intensive care units [5], and our previous work suggests that NINJA could potentially be effective in the adult setting as well [6]. A similar study by Williams et al. further demonstrated the feasibility of this approach in an adult population [7].

NA-AKI prevention systems like NINJA include angiotensin-converting enzyme inhibitors (ACEIs) and angiotensin receptor blockers (ARBs) on their lists of medications with nephrotoxic potential. These agents are known to increase creatinine through inhibition of efferent arteriole vasoconstriction [8], and have been shown to be strong predictors of a creatinine rise ≥ 0.3 mg/dL within NINJA’s high-nephrotoxin exposure population [9]. However, whether ACEI/ARB agents should truly be considered nephrotoxic has become a matter of debate [10, 11]. Some authors have argued that because ACEI/ARBs induce creatinine changes that are likely hemodynamic rather than due to tubular injury, and because subsequent failures to reinitiate ACEI/ARBs after hospitalization have been strongly linked to poor post-discharge outcomes [12], that ACEI/ARBs should not be preemptively discontinued within these AKI prevention programs. Further data regarding AKI development with ACEI/ARB usage, especially in the setting of multiple other nephrotoxins, would help inform whether or not ACEI/ARBs should be included on nephrotoxin lists within AKI prevention systems like NINJA.

In this study we attempt to address the question by evaluating outcomes in patients receiving two non-ACEI/ARB nephrotoxic medications, who would not be considered to have high-nephrotoxin exposure, to patients receiving three including an ACEI/ARB, who would be considered to have high-nephrotoxin exposure due to the additional ACEI/ARB. We hypothesized that ACEI/ARBs would be associated with higher rates of AKI and higher rates of stage 2/3 AKI, which could provide justification for targeting ACEI/ARBs for preemptive discontinuation in these patients.

## Materials and methods

**Study Population**: We obtained local institutional review board approval (HawkIRB 202008447) with a waiver of informed consent based on its retrospective nature. All adult patients ≥ 18 years of age who were admitted to the University of Iowa Hospital from 2014 to 2022 were eligible for inclusion in the analysis. Exclusion criteria were: (1) admissions < 72 h, (2) initial nephrotoxin exposure > 14 days from admission in order to focus on exposures earlier in an admission, (3) initial qualifying nephrotoxin exposure < 48 h prior to hospital discharge, (4) AKI prior to or on the day of initial high nephrotoxin exposure, (5) missing laboratory values or vital sign data, (6) missing baseline creatinine data or baseline creatinine < 0.4 mg/dL, (7) baseline estimated glomerular filtration rate (eGFR) < 25 mL/min/1.73m^2^, and (8) body mass index (BMI) values < 16 or > 150 kg/m^2^. ICU location on the day of initial exposure was an exclusion criterion in the primary analysis, but these patients were included in a secondary analysis.

### Nephrotoxin Exposure Definitions

The NINJA nephrotoxin list [13], which includes 58 medications, was used to determine nephrotoxin exposure for the primary and secondary analyses (Supplemental Table S1). Current NA-AKI prevention programs, including NINJA, incorporate exposure to ≥ 3 simultaneous nephrotoxic agents within their definition of high nephrotoxin exposure. In the primary analysis, we compared patients on two non-ACEI/ARB nephrotoxins to patients on three nephrotoxins, one of which had to be an ACEI/ARB. This allowed us to compare patients who would generally not be counted as having a high nephrotoxin exposure to a group that would, by virtue of being on an ACEI/ARB.

### AKI Definition and Staging

Baseline creatinine was the lowest creatinine value in the six months prior to admission, or the admission creatinine if previous creatinine is not available, as per NINJA convention. AKI was defined as a creatinine increase of 0.3 mg/dL or an increase of 50% from baseline. Stage 2/3 AKI was defined as at least a doubling of creatinine from baseline, or an absolute creatinine value ≥ 4.0 mg/dL [14]. Creatinine change was determined based on the highest creatinine within the specified time period (3, 7, or 14 days) compared to the baseline value, as opposed to a rolling 48 h window. Because creatinine is a lagging marker of injury, AKI events that occurred on the first day of high nephrotoxin exposure were excluded.

### Data Pre-processing

For patient-days missing laboratory or vital sign values, we first forward-filled data, and then backward-filled data to fill all patient-days, as previously described [9], which accounted for 15% of hospital-day laboratory values. For patient-days with multiple laboratory values, the median value was selected. For days with multiple vital sign measurements, the lowest mean arterial pressure and highest patient temperature were selected.

### Variables

The primary outcome was all-stage AKI development, and the secondary outcome was stage 2/3 AKI development. We evaluated for AKI and stage 2/3 AKI at 3 days, 7 days, and 14 days. Covariates used to construct IPTW weights included age, sex, race, body mass index, patient co-morbidities (diabetes, moderate or severe liver disease, coronary artery disease, congestive heart failure, chronic kidney disease, peripheral vascular disease, malignancy history, and cerebrovascular disease, defined by ICD-10 codes using the method of Quan et al. [15]), laboratory values (baseline eGFR, white blood cell count, hemoglobin, platelets, and blood urea nitrogen), vital signs (highest temperature, lowest MAP on the day of exposure), admitting service (categorized as medicine, surgery, cardiac and vascular surgery, neurology/neurosurgery, and other), and the medication classes of the two non-ACEI/ARB nephrotoxins (antibiotics with vancomycin and piperacillin-tazobactam as distinct categories, antivirals, calcineurin inhibitors, chemotherapy, contrast dye, non-steroidal anti-inflammatory drugs (NSAIDs), and other).

### Statistical Analysis

Means and standard deviations or counts and percentages were used to describe the distribution of continuous and categorical variables, respectively. To address confounding, inverse probability of treatment weighting (IPTW) was performed. A multivariable logistic regression model was constructed to estimate the probability of receiving an ACEI/ARB on the initial exposure day using the prespecified baseline covariates. Stabilized average treatment effect (ATE) weights were calculated as the marginal probability of the treatment actually received divided by the predicted probability of receiving that treatment. Covariate balance before and after weighting was assessed using standardized mean differences (SMDs), with values < 0.1 considered indicative of acceptable balance. For longitudinal outcome analyses, generalized estimating equation (GEE) models with a logit link and robust (sandwich) standard errors to account for the IPTW were fitted. Models included IPTW weights, ACEI/ARB status, timepoint, an ACEI/ARB × time interaction term, and any covariates with residual SMD > 0.1 after weighting. Adjusted predicted probabilities were derived from the fitted models at each timepoint for both treatment groups. From these model-based estimates, absolute risk differences and relative risks (with 95% confidence intervals derived using delta methods) were calculated. Time-specific odds ratios were obtained directly from model coefficients. The analysis was then repeated including ICU patients at the time of initial exposure. We conducted one subgroup analysis using the above analysis in patients with chronic kidney disease, defined as a baseline eGFR < 60 mL/min. Additional sensitivity analyses included a Poisson regression model with log link and robust variance estimation to directly estimate relative risks and an ordinal logistic regression model treating AKI as an ordered outcome (no AKI, stage 1 AKI, stage 2/3 AKI). A two-sided p-value of < 0.05 was used throughout for significance. All analyses were conducted using SPSS (IBM Corp. Released 2023. IBM SPSS Statistics for Windows, Version 29.0.2.0 Armonk, NY).

## Results

### Patient Characteristics

**Fig. 1:**
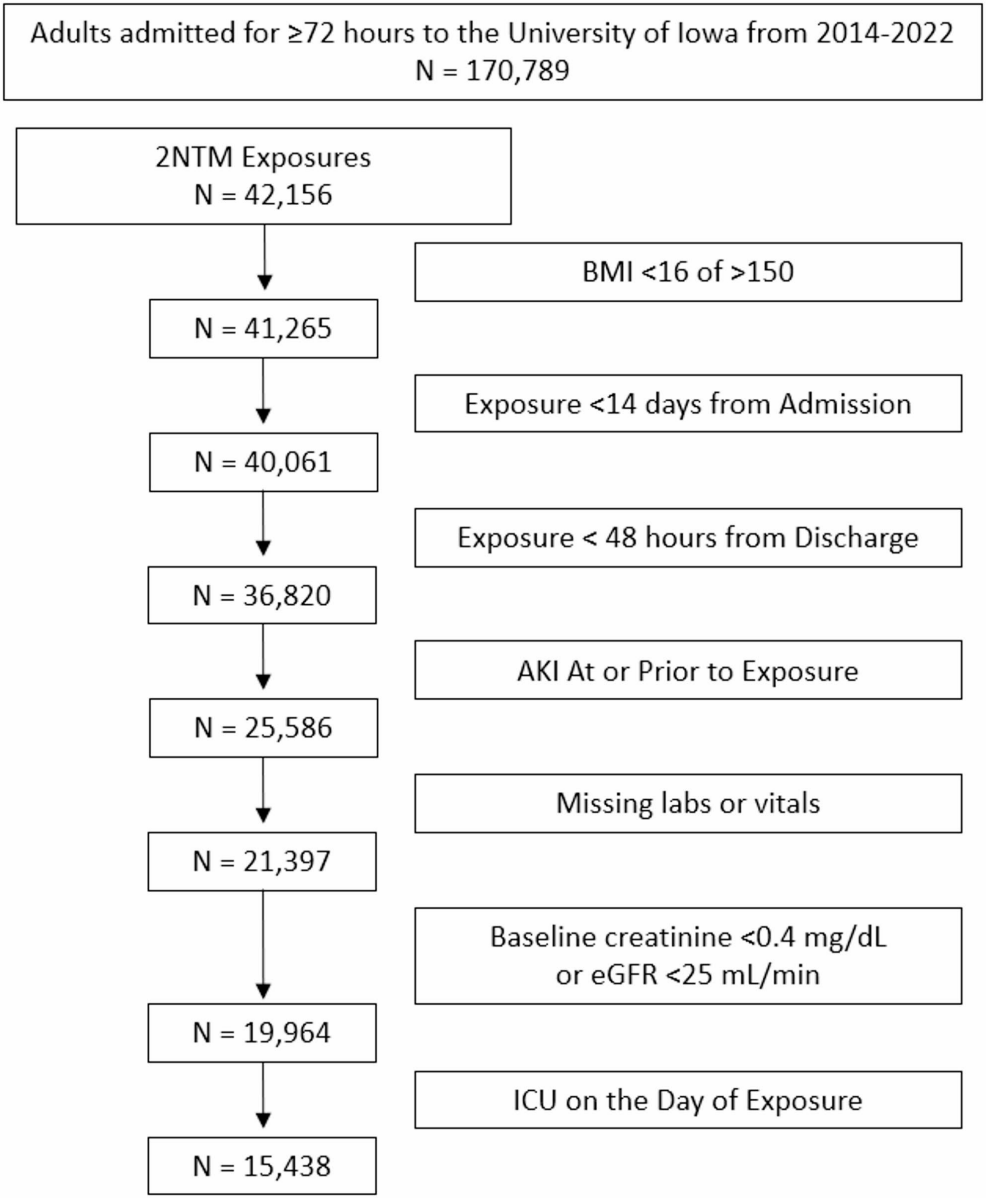
Cohort definition flow diagram. AKI – acute kidney injury; eGFR – estimated glomerular filtration rate; ICU – intensive care unit

We identified 170,789 admissions > 72 h (Fig. 1), of which 42,156 (25%) of admissions received 2 non-ACEI/ARB agents on the NINJA nephrotoxic medication list on the same day. Following exclusions there were 15,438 eligible admissions, of which 1,106 (7.2%) also received an ACEI/ARB. When ICU admissions were included, the population increased to 19,964, of which 1,308 (6.6%) also received an ACEI/ARB.

Baseline clinical characteristics for the primary analysis are given in Table 1, and for the ICU-inclusive analysis in Table 2. Notably, patients receiving ACEI/ARBs were older and had higher comorbidity burdens, including higher proportions of diabetes, coronary artery disease, congestive heart failure, peripheral vascular disease, and cerebrovascular disease. Classes of the other nephrotoxic medications used were similar between groups (Supplemental Figure S1).

**Table 1:** Patient characteristics from the non-ICU population on the day of initial exposure, before and after IPTW weights

Characteristics*	Prior To IPTW Matching			After IPTW Matching		
	ACEI/ARB	Non-ACEI/ARB	SMD	ACEI/ARB	Non-ACEI/ARB	SMD
	(*n* = 1,106)*	(*n* = 14,332)*		*N* = 1,059	*N* = 14,329	
Age (years)	65 (54–73)	59 (44–69)	0.443	62 (49–70)	59 (45–69)	0.142
Female sex	479 (43.3)	7,033 (49.1)	0.116	520 (49.1)	6,974 (48.7)	0.008
Caucasian Race	995 (90.0)	12,789 (89.2)	0.024	961 (90.7)	12,794 (89.3)	0.048
Body mass index (kg/m2)	30 (26–36)	28 (24–34)	0.200	29 (25–35)	28 (24–34)	0.068
**Comorbidities**						
Myocardial Infarction	72 (6.5)	298 (2.1)	0.220	28 (2.6)	343 (2.4)	0.018
Congestive Heart Failure	160 (14.5)	961 (6.7)	0.254	103 (9.7)	1,045 (7.3)	0.086
Diabetes Mellitus	245 (22.2)	1,713 (12.0)	0.274	155 (14.6)	1,820 (12.7)	0.056
COPD	198 (17.9)	2,119 (14.8)	0.084	150 (14.2)	2,150 (15.0)	0.025
Chronic Kidney Disease	86 (7.8)	1,314 (9.2)	0.050	121 (11.4)	1,301 (9.1)	0.078
Liver Disease	50 (4.5)	774 (5.4)	0.041	48 (4.5)	764 (5.3)	0.038
Malignancy History	262 (23.7)	3,882 (27.1)	0.078	298 (28.1)	3,846 (26.8)	0.028
PVD	89 (8.0)	788 (5.5)	0.102	71 (6.7)	816 (5.7)	0.042
Cerebrovascular disease	140 (12.7)	914 (6.4)	0.215	84 (7.9)	982 (6.9)	0.040
**Labs and Vital**,** median (IQR)**						
Baseline eGFR (mL/min)	95 (79–104)	99 (83–112)	0.198	97 (79–107)	99 (83–111)	0.134
WBC (10^9^/L)	9.2 (6.7–12.2)	9.9 (6.8–13.8)	0.087	9.4 (6.8–12.9)	9.9 (6.8–13.7)	0.029
Hemoglobin (g/dL)	11.4 (9.7–13.0)	11.1 (9.3–12.7)	0.134	11.0 (9.3–12.6)	11.1 (9.3–12.7)	0.024
Platelets (10^9^/L)	235 (172–306)	235 (167–318)	0.032	241 (169–314)	235 (168–318)	0.031
BUN (mg/dL)	15 (11–19)	13 (9–18)	0.180	14 (10–18)	13 (9–18)	0.084
Low MAP (mmHg)	80 (71–88)	78 (70–86)	0.116	79 (70–88)	78 (70–87)	0.043
High Temp (Fahrenheit)	99 (98.2–99.5)	99 (98.4–99.5)	0.033	99.1 (98.4–99.7)	99 (98.4–99.5)	0.039
**Admitting Service**			0.355			0.046
Medicine	689 (62.3)	7,188 (50.2)		560 (52.9)	7,314 (51.0)	
Surgery	181 (16.4)	3,517 (24.5)		250 (23.6)	3,432 (23.9)	
Cardio/Vascular Surgery	81 (7.3)	659 (4.6)		58 (5.5)	688 (4.8)	
Neurology/Neurosurgery	103 (9.3)	633 (4.4)		50 (4.7)	683 (4.8)	
Other	181 (4.7)	3,517 (16.3)		140 (13.2)	2,213 (15.4)	
**Medications**						
Antivirals	177 (16.0)	2,303 (16.1)	0.002	170 (16.1)	2,303 (16.1)	0.001
Chemotherapy	37 (3.3)	372 (2.6)	0.044	23 (2.2)	378 (2.6)	0.031
Contrast Dye	653 (59.0)	7,185 (50.1)	0.180	551 (52.0)	7,275 (50.8)	0.025
Immunosuppression	40 (3.6)	406 (2.8)	0.044	41 (3.9)	415 (2.9)	0.053
NSAIDs	285 (25.8)	3,996 (27.9)	0.048	255 (24.1)	3,971 (27.7)	0.082
Other Nephrotoxins	91 (8.2)	817 (5.7)	0.099	62 (5.8)	841 (5.9)	0.002
Pip-Tazo	336 (30.4)	5,253 (36.7)	0.133	418 (39.5)	5,188 (36.2)	0.067
Vancomycin	433 (39.2)	5,217 (36.4)	0.057	413 (39.0)	5,247 (36.6)	0.048
Other Antibiotics	31 (2.8)	863 (6.0)	0.157	40 (3.8)	830 (5.8)	0.095

* Presented as counts and percentages or means and median and interquartile range, respectively

ACEI/ARB – angiotensin converting enzyme inhibitor/angiotensin receptor blocker; BUN – Blood Urea Nitrogen; MAP – Mean Arterial Pressure; NSAID – Non-steroidal Anti-Inflammatory Drug; NTM – Nephrotoxic Medication

**Table 2:** Patient characteristics from the ICU-inclusive population gen on the day of initial exposure, before and after IPTW weights

Characteristics*	Prior To IPTW Matching			After IPTW Matching		
	ACEI/ARB	Non-ACEI/ARB	SMD	ACEI/ARB	Non-ACEI/ARB	SMD
	(*n* = 1,308)*	(*n* = 18,656)*		(*n* = 1,248)	(*n* = 18,651)	
Age (years)	65 (55–73)	59 (45–69)	0.401	61 (50–70)	60 (46–70)	0.134
Female sex	544 (41.6)	8,775 (47.0)	0.110	597 (47.8)	8,708 (46.7)	0.023
Caucasian Race	1,179 (90.1)	16,675 (89.4)	0.025	1,129 (90.4)	16,681 (89.4)	0.033
Body mass index (kg/m2)	30 (26–36)	28 (24–34)	0.185	29 (25–35)	28 (24–34)	0.049
**Comorbidities**						
Myocardial Infarction	93 (7.1)	531 (2.8)	0.197	41 (3.3)	583 (3.1)	0.007
Congestive Heart Failure	194 (14.8)	1,437 (7.7)	0.227	139 (11.1)	1,529 (8.2)	0.099
Diabetes Mellitus	294 (22.5)	2,293 (12.3)	0.271	191 (15.3)	2,421 (13.0)	0.066
COPD	229 (17.5)	2,794 (15.0)	0.069	180 (14.4)	2,823 (15.1)	0.020
Chronic Kidney Disease	104 (8.0)	2,107 (11.3)	0.114	161 (12.9)	2,067 (11.1)	0.056
Liver Disease	60 (4.6)	1,075 (5.8)	0.053	66 (5.3)	1,060 (5.7)	0.017
Malignancy History	283 (21.6)	4,429 (23.7)	0.050	325 (26.0)	4,403 (23.6)	0.057
PVD	125 (9.6)	1,202 (6.4)	0.115	108 (8.7)	1,243 (6.7)	0.076
Cerebrovascular disease	225 (17.2)	1,663 (8.9)	0.248	130 (10.4)	1,769 (9.5)	0.030
**Labs and Vital**,** median (IQR)**						
Baseline eGFR (mL/min)	97 (77–107)	98 (80–110)	0.092	96 (80–105)	98 (80–111)	0.094
WBC (10^9^/L)	9.9 (7.2–13.6)	10.4 (7.2–14.5)	0.117	9.6 (6.9–12.7)	10.5 (7.2–14.6)	0.035
Hemoglobin (g/dL)	11.0 (9.3–12.6)	11.0 (9.2–12.7)	0.163	11.4 (9.7–13.0)	11.0 (9.2–12.6)	0.019
Platelets (10^9^/L)	227 (166–306)	226 (161–308)	0.002	231 (172–301)	226 (160–308)	0.033
BUN (mg/dL)	14 (10–20)	14 (10–19)	0.089	15 (11–20)	14 (10–19)	0.045
Low MAP (mmHg)	78 (68–87)	76 (67–86)	0.192	79 (70–88)	76 (67–85)	0.079
High Temp (Fahrenheit)	99.1 (98.4–99.6)	99 (98.4–99.6)	0.041	99.1 (98.4–99.7)	99 (98.4–99.6)	0.030
**Admitting Service**			0.347			0.040
Medicine	724 (55.4)	8,777 (47.0)		590 (47.3)	8,876 (47.6)	
Surgery	204 (15.6)	4,847 (26.0)		313 (25.1)	4,717 (25.3)	
Cardio/Vascular Surgery	143 (10.9)	1,345 (7.2)		118 (9.5)	1,393 (7.5)	
Neurology/Neurosurgery	184 (14.1)	1,303 (7.0)		93 (7.5)	1,390 (7.5)	
Other	53 (4.1)	2,384 (12.8)		134 (10.7)	2,274 (12.2)	
ICU Unit	202 (15.4)	4,324 (23.2)	0.197	254 (20.3)	4,227 (22.7)	0.057
**Medications**						
Antivirals	186 (14.2)	2,679 (14.4)	0.004	183 (14.7)	2,677 (14.4)	0.010
Chemotherapy	37 (2.8)	381 (2.0)	0.051	22 (1.8)	389 (2.1)	0.025
Contrast Dye	813 (62.2)	9,613 (51.5)	0.216	679 (54.4)	9,743 (52.2)	0.043
Immunosuppression	42 (3.2)	504 (2.7)	0.030	46 (3.7)	511 (2.7)	0.053
NSAIDs	294 (22.5)	4,251 (22.8)	0.007	250 (20.0)	4,245 (22.8)	0.066
Other Nephrotoxins	99 (7.6)	931 (5.0)	0.106	65 (5.2)	961 (5.2)	0.003
Pip-Tazo	413 (31.6)	7,422 (39.8)	0.172	511 (40.9)	7,320 (39.2)	0.034
Vancomycin	537 (41.1)	7,985 (42.8)	0.035	530 (42.5)	7,963 (42.7)	0.004
Other Antibiotics	35 (2.7)	1,078 (5.8)	0.155	51 (4.1)	1,040 (5.6)	0.068

* Presented as counts and percentages or means and median and interquartile range, respectively

ACEI/ARB – angiotensin converting enzyme inhibitor/angiotensin receptor blocker; BUN – Blood Urea Nitrogen; MAP – Mean Arterial Pressure; NSAID – Non-steroidal Anti-Inflammatory Drug; NTM – Nephrotoxic Medication

### IPTW Outcomes

In the primary analysis, all covariates achieved standardized mean differences (SMD) < 0.1 after weighting, except for age and baseline eGFR, which were subsequently included as covariates in the weighted GEE model (Supplemental Figure S2). In the ICU-inclusive analysis, only age remained imbalanced (SMD > 0.1). Stabilized weights had a mean of 1.00 (SD 0.23) with a range of 0.12–8.23, indicating adequate overlap and no evidence of extreme weight instability. The overall effective sample size (ESS) was 14,773, corresponding to a variance inflation factor (VIF) of 1.045 (4.5% increase in variance). Group-specific ESS estimates were ESS 647 (VIF 1.71) for ACEI/ARB, and ESS 14,262 (VIF 1.005) for the comparator group. Weight distributions by exposure group are shown in Supplemental Figure S3.

### AKI Outcomes

The adjusted estimated risk of AKI within 7 days for patients receiving ACEI/ARBs was 25% (22–29%), compared to 20% (19–21%) in comparators (adjusted odds ratio (aOR 1.35, 95% CI 1.07–1.71) (Table 3). The risk difference was statistically significant at all timepoints, although the magnitude was greatest at Day 3. In contrast, estimated risks of stage 2/3 AKI were not different at any timepoint, and relative risks (RR) and aOR were not statistically or clinically significant. When the analyses were expanded to include ICU patients, estimated risk of AKI and stage 2/3 AKI increased in both groups (Table 3), but modeling results were similar to the non-ICU analysis at all time points. E-values for the primary analysis ranged from 1.74 to 2.37 for point estimates and 1.34 to 1.90 for the lower confidence limits, and similar E-values were observed in the ICU-inclusive analysis (range 1.76–2.24 for point estimates) (Supplemental Table S2).

**Table 3:** IPTW-adjusted AKI outcomes in patients receiving ACEI/ARB compared to those not receiving, with/without inclusiong of initial exposures in the ICU setting

Outcome	Time	ACE/ARB Risk %, (95% CI)	No ACE/ARB Risk %, (95% CI)	Risk Difference % (95% CI)	Relative Risk (95% CI)	Odds Ratio (95% CI)
**General Wards**						
All AKI	3 Days	21 (18–25)	14 (14–15)	7 (3.6–10.4)	1.50 (1.29–1.74)	1.66 (1.27–2.16)
	7 Days	25 (22–29)	20 (19–21)	5 (1.6–8.4)	1.25 (1.10–1.43)	1.35 (1.07–1.71)
	14 Day	28 (25–32)	23 (23–24)	5 (1.4–8.6)	1.22 (1.07–1.39)	1.31 (1.10–1.56)
AKI Stage 2/3	3 Days	2 (1–3)	2 (2–2)	0 (− 0.8–0.8)	1.00 (0.66–1.52)	0.91 (0.64–1.42)
	7 Days	4 (3–5)	4 (3–4)	0 (− 1.2–1.2)	1.00 (0.77–1.30)	1.14 (0.90–1.54)
	14 Day	4 (3–6)	5 (4–5)	−1 (− 2.4–0.4)	0.80 (0.58–1.10)	0.92 (0.66–1.28)
**Including ICU**						
All AKI	3 Days	23 (19–26)	16 (16–17)	7 (3.6–10.4)	1.44 (1.23–1.68)	1.66 (1.27–2.17)
	7 Days	29 (26–33)	23 (23–24)	6 (2.6–9.4)	1.26 (1.11–1.43)	1.36 (1.09–1.70)
	14 Day	34 (30–37)	27 (27–28)	7 (3.3–10.7)	1.26 (1.13–1.41)	1.35 (1.14–1.60)
AKI Stage 2/3	3 Days	3 (2–5)	3 (3–3)	0 (− 1.4–1.4)	1.00 (0.66–1.53)	0.85 (0.57–1.49)
	7 Days	6 (4–8)	5 (4–5)	1 (− 1.9–3.9)	1.20 (0.85–1.70)	1.18 (0.68–1.49)
	14 Day	8 (6–10)	6 (6–6)	2 (− 1.1–5.1)	1.33 (0.99–1.79)	1.25 (0.91–1.73)

### Sensitivity and Subgroup Analyses

When using the Poisson regression models with covariates rather than IPTW, relative risks were similar to those obtained using GEE at all timepoints (aRR of AKI by day 7 was 1.16 (1.04–1.29) (Supplemental Table S3), and for AKI 2/3 was 1.12 (0.83–1.51)). In the ordinal regression model, ACEI/ARB initiation was associated with higher odds of being in a more severe AKI category at day 7 (ordinal OR 1.30, *p* < 0.001), but Test of Parallel Lines was significant at *p* = 0.04. This finding was consistent with the binary analyses indicating that the observed association appeared to be driven primarily by increases in stage 1 AKI rather than stage 2/3 events. Finally, the CKD subgroup analysis showed higher rates of AKI in both groups with higher aOR and aRR for AKI association with ACEI/ARB use, but were not statistically significant (Supplemental Table S4).

## Discussion

In this retrospective study we compared patients receiving 2 non-ACEI/ARB nephrotoxic medications to 2 plus an ACEI/ARB, which allowed us to compare patients who generally would not be considered to have high nephrotoxin exposure to those who would, by virtue of the additional ACEI/ARB agent. ACEI/ARB exposure was associated with higher risk of AKI overall, but not with higher risk of stage 2/3 AKI. These findings were consistent when expanded to include ICU patients. These results suggest that discontinuation of ACEI/ARB agents within NA-AKI prevention efforts like NINJA and others might be effective in reducing stage 1 AKI, but would be unlikely to meaningfully impact rates of stage 2/3 AKI in adults.

ACEI/ARB agents have long been known to increase creatinine primarily through a reduction in glomerular pressure due to inhibition of efferent arteriole vasoconstriction, not necessarily due to tubular injury [16]. Stopping ACEI/ARBs in hospitalized patients with any degree of AKI is still common and often recommended [17], but skeptics of ACEI/ARB discontinuation have argued: (1) small changes in creatinine in hospitalized patients with ACEI/ARB may be due to effects on the efferent arteriole rather than true tubular injury, (2) ACEI/ARBs may protect against the development of CKD following AKI [18, 19], and (3) ACEI/ARBs are frequently not restarted after being discontinued in the hospital, and patients therefore do not receive the long-term benefits of these medications [20–22]. For example, in a study of nearly 50,000 patients whose hospitalization featured an episode of AKI, only 48% of patients were prescribed an ACEI/ARB at 6 months from discharge. For those starting a new ACEI/ARB, there was a 15% reduction in adjusted hazards for mortality at 2 years, and for those continuing a previous ACEI/ARB, there was a 23% reduction^12^.

Our aim in this paper was to investigate the associations of ACEI/ARB usage and AKI development, particularly development of stage 2/3 AKI, to help inform decisions regarding whether these agents should be included in future iterations of NINJA or other NA-AKI prevention systems. Previous studies have shown that nearly all patients with higher stages of AKI had elevated AKI biomarker and histologic evidence of injury on biopsies, compared to about 50% in stage 1 AKI [23]. It is therefore unlikely that higher stages of AKI can be attributed to hemodynamic changes alone. In this paper, the aORs for stage 2/3 AKI were near 1, with inconsistent directionality of effect at different timepoints, suggesting a lack of association with ACEI/ARB use. Lack of significance may have derived from the relatively low rates of stage 2/3 AKI, especially in the non-ICU group (3.5%), but our findings would be consistent with theoretical expectations for a medication that raises creatinine through hemodynamic effects rather than true structural injury. Additional studies are needed with larger cohorts and ideally incorporating novel AKI biomarkers would help clarify the proportion of stage 1 events attributable to hemodynamic versus structural mechanisms.

Rates of ACEI/ARB use were higher than anticipated in this at-risk population, at 7.2% and 6.6% of cases in the general wards and ICUs, respectively. About 85% of patients received their ACEI/ARBs for multiple days, and the majority (71%) of ACEI/ARB users were still on these agents when AKI developed. We therefore think it unlikely that early cessation of ACEI/ARB explains the absence of association with stage 2/3 AKI, although this possibility cannot be ruled out.

There have been a number of prospective studies that have attempted to improve outcomes through discontinuation of ACEI/ARBs after AKI development, and while several have shown reductions in nephrotoxin prescribing, none have been successful in reducing progression to higher stages of AKI or otherwise preventing AKI-related morbidity and mortality [24–30]. A recent prospective randomized-controlled trial including over 5,000 participants compared an electronic alert in patients with AKI on nephrotoxic agents including ACEI/ARBs. These agents were discontinued at a higher rate in the alert group, but there was no difference in the composite outcome of progression of AKI, dialysis, or death within 14 days compared to usual care [26]. In contrast, preventive systems like NINJA have been shown to reduce NA-AKI [4, 31]. These findings align with our observation that ACEi/ARB exposure may increase stage 1 AKI incidence without influencing progression to more severe injury.

There are several limitations inherent within our analysis. As an observational study, residual confounding cannot be excluded and unmeasured factors such as need for pressors in ICU patients or clinical indication for ACEi/ARB initiation could attenuate the observed associations. Given the relatively low number of Stage 2/3 AKI events in the ACEI/ARB groups, we may have been underpowered for this secondary outcome. We could not adequately account for discontinuation of ACEI/ARB agents after stage 1 AKI development. In addition, the NINJA nephrotoxin list is not exhaustive, and some patients with high nephrotoxic burdens may therefore have been unrecognized in this analysis. This analysis could not account for time-dependent changes in labs, vitals, and medication usage. Use of IPTW did leave some residual imbalance in age and eGFR, which we tried to address by including these values in the GEE model. Finally, we did not have access to oupatient prescribing data in this hospital database, so we couldn’t include ACEI/ARB usage before or after the admission into our analyses.

In conclusion, among hospitalized adults receiving two nephrotoxic medications, addition of an ACEi/ARB was associated with increased incidence of AKI overall but not with higher rates of stage 2/3 AKI. These findings suggest that discontinuation of these drugs pre-emptively might lower stage 1 AKI, but are less likely to reduce stage 2/3 AKI. Any potential short-term benefit of discontinuation should be carefully weighed against the established long-term cardiovascular and renal benefits of these agents, particularly if they are not resumed after discharge.

## Supplementary Information

Below is the link to the electronic supplementary material.

Supplementary Material 1

## Data Availability

The data that support the findings of this study are available from the University of Iowa but restrictions apply to the availability of these data, which were used under approval for the current study, and so are not publicly available. Data are however available from the authors upon reasonable request and with permission of the University of Iowa.
