# Supplementary material for: The impacts of angiotensin converting enzyme inhibitors and angiotensin receptor blockers on rates of acute kidney injury in hospitalized adults receiving multiple nephrotoxins
Source: BMC Nephrol. 2026 Mar 26;27:281. doi: 10.1186/s12882-026-04884-3 (PMC13141392; doi:10.1186/s12882-026-04884-3)

**Supplemental Table S1.** Medications within NINJA, by class. These medications were used to define high nephrotoxin exposure in the 2 Nephrotoxic Medications (2NTM) analyses.

| **Drug Class** | **Drug Name** | |
| --- | --- | --- |
| ACEI/ARB | Captopril  Enalapril  Enalaprilat | Lisinopril  Losartan  Valsartan |
| Antibiotics | Ambisome  Amikacin  Amphotericin B  Clavulanic Acid  Colistimethate  Gentamicin  Nafcillin | Pentamadine  Piperacillin  Polymixin B  Tazobactam  Ticarcillin  Tobramycin  Vancomycin |
| Antivirals | Acyclovir  Cidofovir  Foscarnet  Ganciclovir | Tenofovir  Valacyclovir  Valganciclovir |
| Chemotherapies | Carboplatin  Cisplatin  Ifosfamide | Methotrexate  Mitomycin |
| Immunosuppression | Cyclosporine  Sirolimus | Tacrolimus |
| Iodinated Contrast Dye | Diatrizoate meglumine  Diatrizoate sodium  Iodixanol  Iohexol  Iopamidol | Iopromide  Ioversol  Ioxaglate meglumine  Ioxilan |
| NSAIDs | Celecoxib  Ibuprofen  Indomethacin | Ketorolac  Naproxen |
| Others | Deferasirox  Lithium  Mesalamine  Pamidronate | Sulfasalazine  Topiramate  Zoledronic acid  Zonisamide |

ACEI – angiotensin converting enzyme inhibitor; ARB – angiotensin receptor blocker;

NSAIDs – non-steroidal anti-inflammatory drugs

**Supplemental Table S2.** E-values for Adjusted Risk Ratios, which represent the minimum strength of unmeasured confounding required to attenuate the observed association to the null.

| **Analysis** | **Time** | **RR** | **Lower 95% CI** | **E-value (RR)** | **E-value (Lower CI)** |
| --- | --- | --- | --- | --- | --- |
| **Primary**  **All AKI** | Day 3 | 1.50 | 1.29 | 2.37 | 1.90 |
|  | Day 7 | 1.25 | 1.10 | 1.81 | 1.43 |
|  | Day 14 | 1.22 | 1.07 | 1.74 | 1.34 |
| **ICU-inclusive**  **All AKI** | Day 3 | 1.44 | 1.23 | 2.24 | 1.76 |
|  | Day 7 | 1.26 | 1.11 | 1.83 | 1.46 |
|  | Day 14 | 1.26 | 1.13 | 1.83 | 1.51 |

**Supplemental Table S3.** Risk ratios obtained using Poisson log-link model, with outcome of any-stage AKI within 7 days of exposure.

| **Characteristics*** | **Any-stage AKI** |
| --- | --- |
| Age (years) | 1.002 (0.999-1.004) |
| Male sex | 1.15 (1.08-1.23) |
| Caucasian Race | 0.93 (0.84-1.03) |
| Body mass index (kg/m2) | 1.005 (1.001-1.008) |
| **Comorbidities** |  |
| Myocardial Infarction | 1.00 (0.84-1.18) |
| Congestive Heart Failure | 1.29 (1.18-1.42) |
| Diabetes Mellitus | 1.08 (0.99-1.17) |
| COPD | 1.08 (0.99-1.17) |
| Chronic Kidney Disease | 1.07 (0.94-1.22) |
| Liver Disease | 1.39 (1.24-1.55) |
| Malignancy History | 1.03 (0.96-1.11) |
| PVD | 1.13 (1.01-1.26) |
| Cerebrovascular disease | 1.04 (0.93-1.17) |
| **Labs and Vital, median (IQR)** |  |
| Baseline eGFR (mL/min) | 0.996 (0.994-0.999) |
| WBC (10^9^/L) | 0.999 (0.996-1.002) |
| Hemoglobin (g/dL) | 0.95 (0.94-0.97) |
| Platelets (10^9^/L) | 1.00 (1.00-1.00) |
| BUN (mg/dL) | 1.009 (1.006-1.013) |
| Low MAP (mmHg) | 1.005 (1.002-1.007) |
| High Temp (Fahrenheit) | 1.002 (0.996-1.007) |
| **Admitting Service** |  |
| Medicine | 1.13 (1.01-1.26) |
| Surgery | 1.01 (0.90-1.13) |
| Cardio/Vascular Surgery | 1.49 (1.29-1.73) |
| Neurology/Neurosurgery | 0.96 (0.79-1.17) |
| Other | Reference |
| **Medications** |  |
| ACEI/ARB | 1.16 (1.04-1.29) |
| Antivirals | 1.79 (1.53-2.09) |
| Chemotherapy | 2.08 (1.72-2.51) |
| Contrast Dye | 1.23 (1.09-1.39) |
| Immunosuppression | 1.49 (1.24-1.78) |
| NSAIDs | 1.08 (0.93-1.26) |
| Other Nephrotoxins | 1.24 (1.03-1.49) |
| Pip-Tazo | 1.89 (1.67-2.14) |
| Vancomycin | 1.53 (1.36-1.72) |
| Other Antibiotics | 1.61 (1.35-1.91) |

**Supplemental Table S4.** Subgroup analysis in patients with chronic kidney disease, IPTW-adjusted AKI outcomes in patients receiving ACEI/ARB compared to those not receiving,

| **Outcome** | **Time** | **ACE/ARB**  **%, (95% CI)** | **No ACE/ARB**  **%, (95% CI)** | **Risk Difference %, (95% CI)** | **Relative Risk**  **(95% CI)** | **Odds Ratio**  **(95% CI)** |
| --- | --- | --- | --- | --- | --- | --- |
| **General Wards** | | | | | | |
| **Primary**  **All AKI** | 3 Days | 38 (26–52) | 27 (24–29) | +11 (−2 to 24) | 1.41 (0.99–2.01) | 1.72 (0.80–3.69) |
|  | 7 Days | 43 (31–56) | 35 (32–37) | +8 (−6 to 22) | 1.23 (0.94–1.61) | 1.46 (0.76–2.80) |
|  | 14 Day | 44 (32–58) | 38 (36–41) | +6 (−8 to 20) | 1.16 (0.89–1.51) | 1.28 (0.74–2.23) |

**Supplemental Figure S1.** Other nephrotoxins used in the 2 Nephrotoxic Medications (2NTM) primary analysis, prior to IPTW.

**Supplemental Figure S2.** Love charts showing SMDs for each variable before and after application of IPTW weights, for the A. Primary analysis and B. ICU-inclusive analysis.


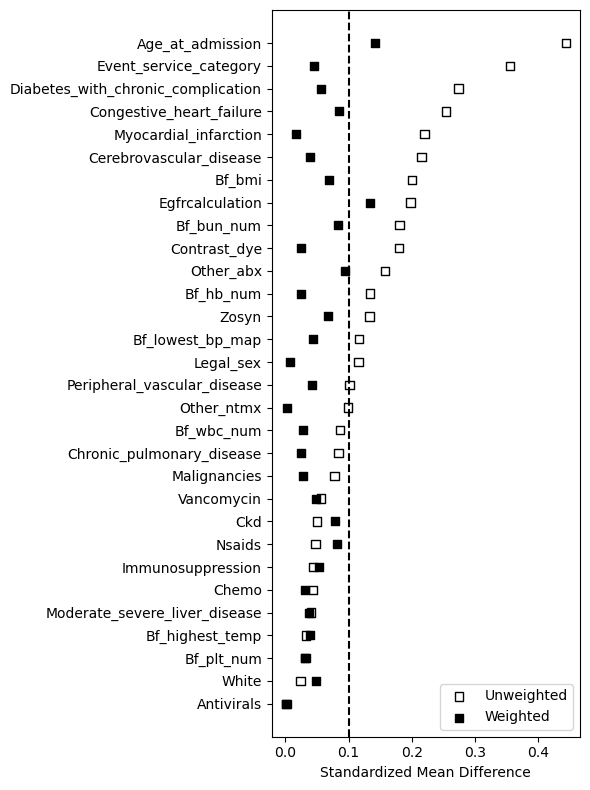


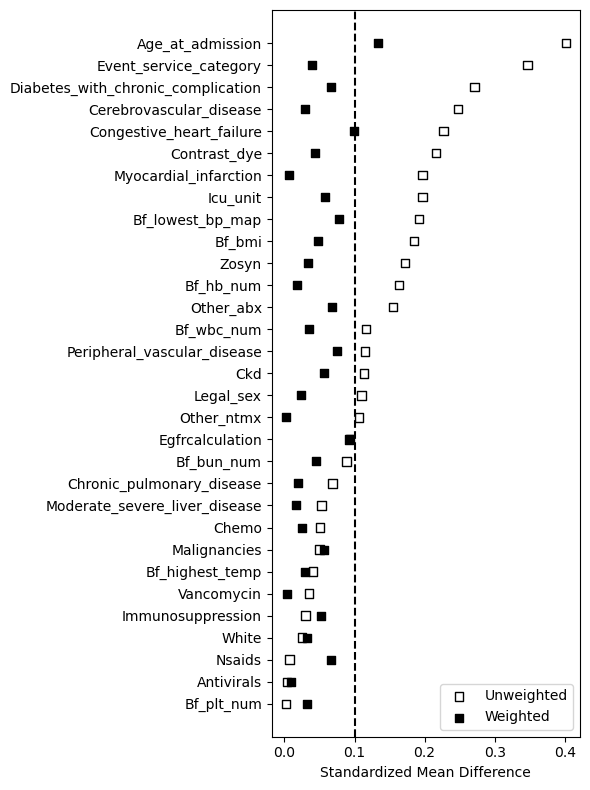


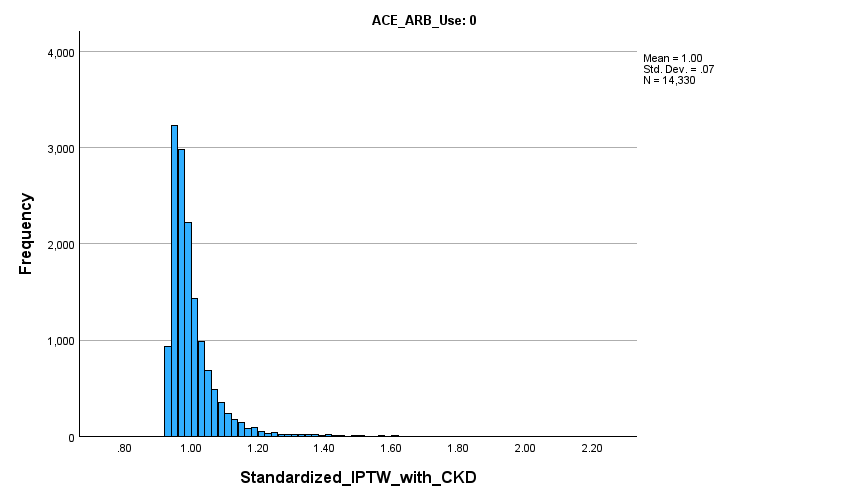
**Supplemental Figure S3.** IPTW distribution of weights for the ACEI/ARB group and non-ACEI/ARB groups.


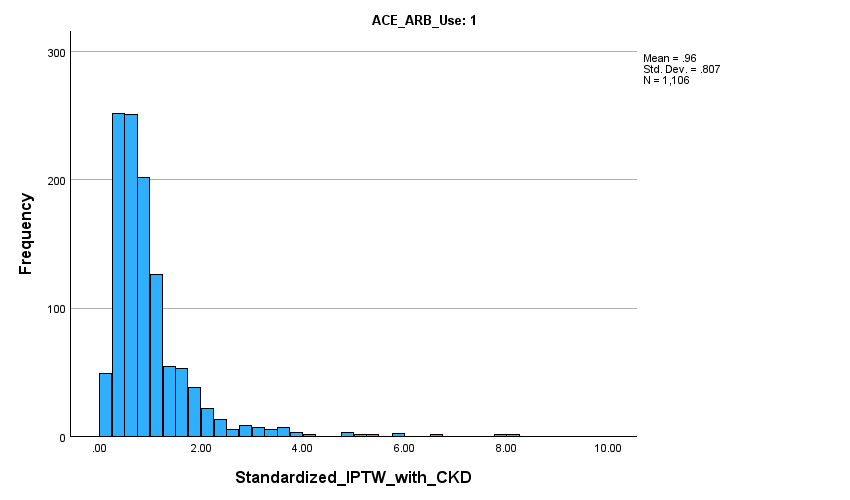

Supplement: Supplementary file 1 — Supplementary Material 1 [file 12882_2026_4884_MOESM1_ESM.docx]
